# Prognostic Significance of Blood Pressure Variability on Beat-to-Beat Monitoring After Transient Ischemic Attack and Stroke

**DOI:** 10.1161/STROKEAHA.117.019107

**Published:** 2017-12-11

**Authors:** Alastair J.S. Webb, Sara Mazzucco, Linxin Li, Peter M. Rothwell

**Affiliations:** From the Department of Clinical Neurosciences, Centre for Prevention of Stroke and Dementia, University of Oxford, United Kingdom.

**Keywords:** cardiovascular diseases, humans, hypertension, risk factors, stroke

## Abstract

**Background and Purpose—:**

Visit-to-visit and day-to-day blood pressure (BP) variability (BPV) predict an increased risk of cardiovascular events but only reflect 1 form of BPV. Beat-to-beat BPV can be rapidly assessed and might also be predictive.

**Methods—:**

In consecutive patients within 6 weeks of transient ischemic attack or nondisabling stroke (Oxford Vascular Study), BPV (coefficient of variation) was measured beat-to-beat for 5 minutes (Finometer), day-to-day for 1 week on home monitoring (3 readings, 3× daily), and on awake ambulatory BP monitoring. BPV after 1-month standard treatment was related (Cox proportional hazards) to recurrent stroke and cardiovascular events for 2 to 5 years, adjusted for mean systolic BP.

**Results—:**

Among 520 patients, 26 had inadequate beat-to-beat recordings, and 22 patients were in atrial fibrillation. Four hundred five patients had all forms of monitoring. Beat-to-beat BPV predicted recurrent stroke and cardiovascular events independently of mean systolic BP (hazard ratio per group SD, stroke: 1.47 [1.12–1.91]; *P*=0.005; cardiovascular events: 1.41 [1.08–1.83]; *P*=0.01), including after adjustment for age and sex (stroke: 1.47 [1.12–1.92]; *P*=0.005) and all risk factors (1.40 [1.00–1.94]; *P*=0.047). Day-to-day BPV was less strongly associated with stroke (adjusted hazard ratio, 1.29 [0.97–1.71]; *P*=0.08) but similarly with cardiovascular events (1.41 [1.09–1.83]; *P*=0.009). BPV on awake ambulatory BP monitoring was nonpredictive (stroke: 0.89 [0.59–1.35]; *P*=0.59; cardiovascular events: 1.08 [0.77–1.52]; *P*=0.65). Despite a weak correlation (*r*=0.119; *P*=0.02), beat-to-beat BPV was associated with risk of recurrent stroke independently of day-to-day BPV (1.41 [1.05–1.90]; *P*=0.02).

**Conclusions—:**

Beat-to-beat BPV predicted recurrent stroke and cardiovascular events, independently of mean systolic BP and risk factors but short-term BPV on ambulatory BP monitoring did not. Beat-to-beat BPV may be a useful additional marker of cardiovascular risk.

## Abstract 2

Supplemental Digital Content is available in the text.

Patients with episodic hypertension in clinic after a previous transient ischemic attack or stroke have a high risk of recurrent stroke,^1,2^ residual visit-to-visit variability in blood pressure (BP) on antihypertensive treatment has a poor prognosis, despite good control of mean BP,^3^ and benefits of some antihypertensive drugs in the prevention of stroke may partly result from reduced variability in systolic BP (SBP).^3,4^ Home day-to-day BP variability (home BP monitoring [HBPM] BPV) is similarly associated with an increased stroke risk,^5,6^ particularly for variability in morning BP^6^ and is reduced by similar medications. In contrast, short-term BPV on awake ambulatory BP monitoring (ABPM) is only weakly predictive of cardiovascular events,^2^ as is within-visit variability in office BP, with short-term BPV also correlating poorly with visit-to-visit BPV.^1,2^ However, the predictive value of beat-to-beat BPV for 5 minutes has not been determined.

Beat-to-beat BPV for 5 minutes is only weakly correlated with day-to-day BPV on HBPM and premorbid visit-to-visit BPV but shares the same physiological associations, suggestive of a similar pathophysiology.^7^ Increased beat-to-beat BPV^8^ and diminished baroreceptor sensitivity (derived from beat-to-beat BP monitoring)^9^ are potentially associated with a worse outcome after a major acute stroke and may be associated with an increased risk of recurrent events.^8^ However, previous studies were small with significant methodological problems. Therefore, we determined the predictive value of beat-to-beat BPV in a prospective cohort of patients with recent transient ischemic attack or minor stroke.

## Materials and Methods

Requests for access to the data and analysis tools in this article will be openly considered. Please contact P.M.R. for further information.

### Study Population

Consecutive patients were recruited between September 2010 and 2015 from the OXVASC (Oxford Vascular Study)^10^ transient ischemic attack and minor stroke clinic. The OXVASC population consists of 92 728 individuals registered with 100 primary-care physicians in Oxfordshire, United Kingdom.^10^ All consenting patients underwent a standardized medical history and examination, ECG, blood tests, and a stroke protocol magnetic resonance imaging brain and contrast-enhanced magnetic resonance angiography (or CT brain and carotid Doppler ultrasound or CT angiogram), an echocardiogram, and 5-day ambulatory cardiac monitoring. All patients were reviewed by a study physician, the diagnosis verified by the senior study neurologist (P.M.R.), etiology determined by a panel of stroke neurologists, and were followed-up face-to-face at 1, 3, 6, and 12 months, and ≤2, 5, or 10 years. Recurrent events were determined at face-to-face follow-up and by multiple overlapping methods of ascertainment, including daily review of hospital admissions, review of death certificates and coroner’s records, manual review of general practitioner records, and linkage to hospital event statistics and death registries.

Participants were excluded if they were <18 years of age, cognitively impaired (Mini-Mental State Examination<23), pregnant; had a recent myocardial infarction, unstable angina, heart failure (New York Heart Association, 3–4 or ejection fraction, <40%), or untreated bilateral carotid stenosis (>70%); and if they had atrial fibrillation during testing. The study was approved by the Oxfordshire Research Ethics Committee.

### BP Measurement

Two sitting clinic BPs, 5 minutes apart, were measured at ascertainment and 1 month in the nondominant arm, by trained personnel after 5 minutes of rest. From ascertainment, patients agreeing to perform HBPM performed 3 home readings for 10 minutes, 3× daily (after waking, midmorning, and evening) with a Bluetooth-enabled, regularly calibrated telemetric IEM Stabil-o-Graph or A&D UA-767 BT. Patients were instructed to relax in a chair for 5 minutes before measuring BP in the nondominant arm or the higher-reading arm when the mean SBP differed by >20 mm Hg. Anonymized measures were securely transmitted via Bluetooth radio and a mobile phone to a password-protected website (t+ Medical, Abingdon, United Kingdom) and medication prescribed according to guidelines,^11^ most frequently with perindopril, indapamide, or amlodipine, to a target of <130/80. The day before the 1-month follow-up, ABPM was performed with an A&D TM-2430 monitor in the nondominant arm. BP was measured every 30 minutes during the day and 60 minutes at night.

Beat-to-beat BPV was measured for 5 minutes at the ascertainment visit or 1-month clinic in a quiet, dimly lit, temperature-controlled room (21–23°C). Continuous 3-lead ECG and finger arterial BP were acquired at 200 Hz (Finometer MIDI) via a Powerlab 8/35 (ADInstruments), from the nondominant arm when possible. Automated calibration was performed until the recording was stable, but turned off during each test, and readings calibrated offline to the mean of 2 supine, oscillometric brachial readings.

### Analysis

BPV on beat-to-beat monitoring was calculated for 5 minutes. Ectopic beats and artefacts were automatically detected, visually reviewed, and removed by linear interpolation. Patients in atrial fibrillation during the recording were excluded. Variability was calculated as the coefficient of variation (CV) about a linear regression across 5 minutes to remove drift in the waveform (residual CV). HBPM variability was derived from the last 7 days of recording before the 1-month follow-up visit, from the average SBP or diastolic BP (DBP) calculated from the last 2 readings of each cluster of 3. Awake BPV on ABPM was derived after automated and manual exclusion of artefacts according to standard criteria.^12^ BPV was derived as the CV (CV=SD/mean) and the residual CV about a moving average on HBPM. Reproducibility of BPV on HBPM was determined in 100 patients between the second and third weeks of monitoring as Pearson *r* and intraclass correlation coefficient. In 50 patients, beat-to-beat BPV was measured at baseline and the 1-month visit according to the same protocol to determine reproducibility of measurement by Pearson *r* and the intraclass correlation coefficient.

Risk of recurrent cardiovascular events was determined per unit increase in mean and variability in SBP or DBP and per SD for the population for each method of measurement by Cox proportional hazards regression, with and without adjustment for age, sex, and major cardiovascular risk factors (hypertension, diabetes mellitus, family history, smoking, atrial fibrillation, and dyslipidemia), and in combined models adjusting for other measures of BPV. The effect of adjustment of beat-to-beat and day-to-day BPV for regression to the mean was estimated by scaling the difference between the mean BPV for each quartile of BPV and the population mean by the intraclass correlation coefficient.^13^

### Literature Review

Pubmed and EMBASE were searched from inception until March 1, 2017, with the terms (“blood pressure” OR “BP” OR “hypertension” OR “BPV” OR “baroreflex” OR “BRS” OR “baroreflex sensitivity”) AND (“stroke” OR “cerebr*” OR “prognosis” OR “death” OR “mortality” OR “cerebrovascular accident” OR “cerebrovascular event” OR “cerebrovascular” OR “leukoaraiosis” OR “white matter hyperintensities” OR “white matter disease” OR “small vessel disease”). All articles reporting recurrent cardiovascular events per unit of beat-to-beat BPV were identified.

## Results

Of 520 patients, 26 had poor-quality recordings because of excessive ectopics or poor-quality finometer recordings because of poor peripheral circulation, whereas 22 were excluded from beat-to-beat analyses because of atrial fibrillation during the recording, which limits the accuracy of BPV measurement, leaving 472 patients with valid beat-to-beat recordings. Four hundred sixty-six of 520 patients had adequate HBPM (2.9 readings per cluster for median 29 days) and 461 of 520 had adequate ABPM (Table 1), with 405 patients with adequate monitoring undergoing all forms of recording. There were weak-positive associations between BPV measured with different methods (beat-to-beat CV versus HBPM residual CV: *r*=0.119, *P*=0.017; beat-to-beat CV versus awake SBP CV: *r*=0.04, *P*=0.37; HBPM residual CV versus awake SBP CV: *r*=0.20, *P*<0.001) but limited associations with demographic variables (Table 1).

**Table 1.**
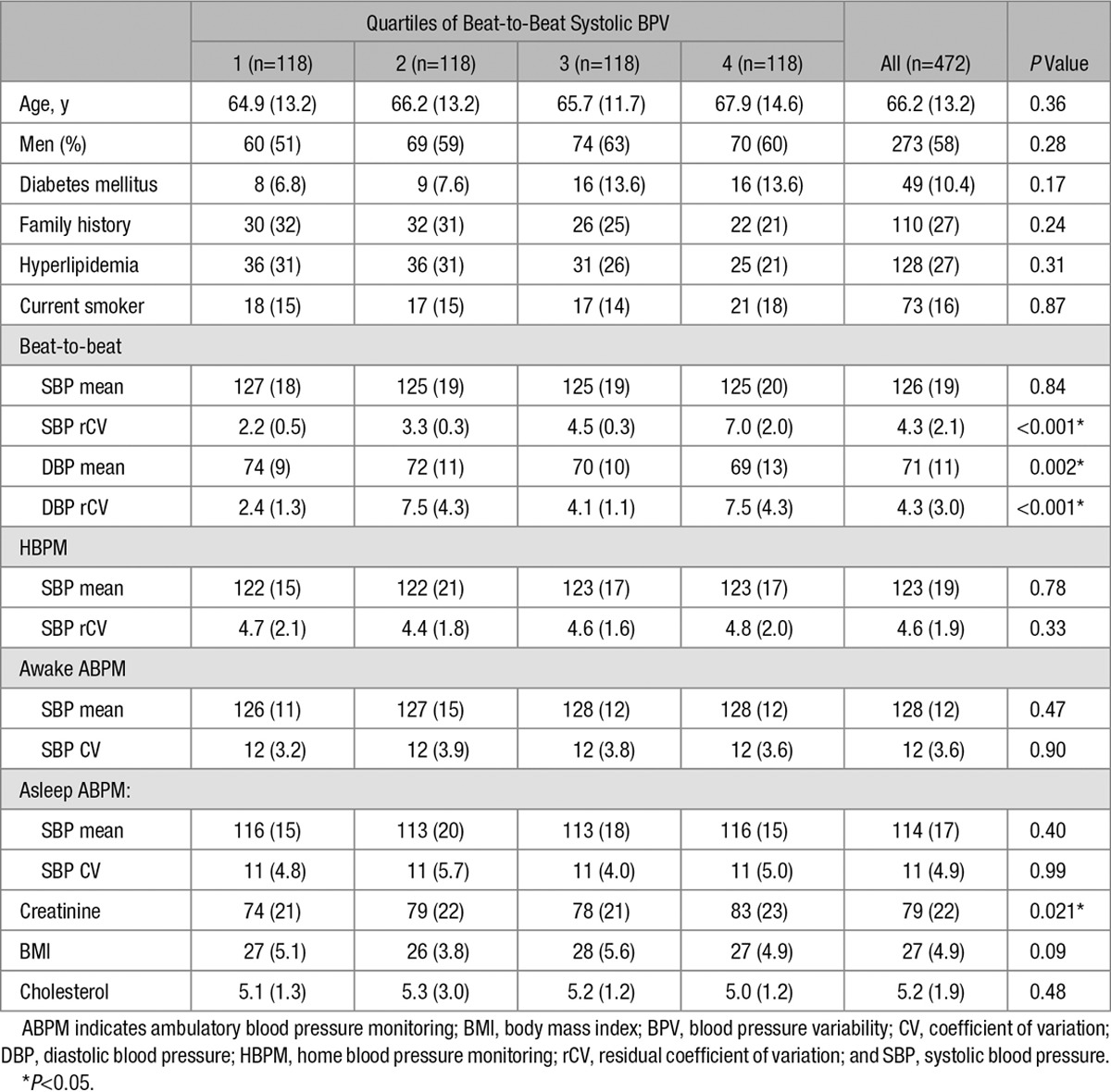
Demographics of 472 Patients With Adequate Beat-to-Beat Recording in Sinus Rhythm During the Recording

BPV on beat-to-beat monitoring in the 405 patients undergoing all forms of monitoring was associated with an increased risk of ischemic stroke, any stroke, and all cardiovascular events, independently of mean SBP (Table 2), before and after adjustment for age and sex, with a significant association with the risk of recurrent ischemic stroke remaining after adjustment for other cardiovascular risk factors (hazard ratio per SD, 1.40 [1.00–1.94]; *P*=0.047). Relationships were similar for all patients undergoing each form of monitoring and largely unchanged by adjustment for mean SBP (Table I in the online-only Data Supplement). The hazard ratio per 1% increase in beat-to-beat CV for stroke was 1.24 (1.07–1.43; *P*=0.004; Table II in the online-only Data Supplement). BPV on home monitoring was not as strongly associated with stroke risk but was associated with all-cause mortality and a composite of death and cardiovascular events (Table 2). Beat-to-beat DBP variability was not predictive of recurrent events, although home DBP variability weakly predicted all-cause mortality (Table III in the online-only Data Supplement). In contrast to beat-to-beat and home monitoring, BPV on ABPM did not predict any recurrent events (Table 2), but mean SBP on all 3 methods of measurement predicted the risk of future events (Table IV in the online-only Data Supplement).

**Table 2.**
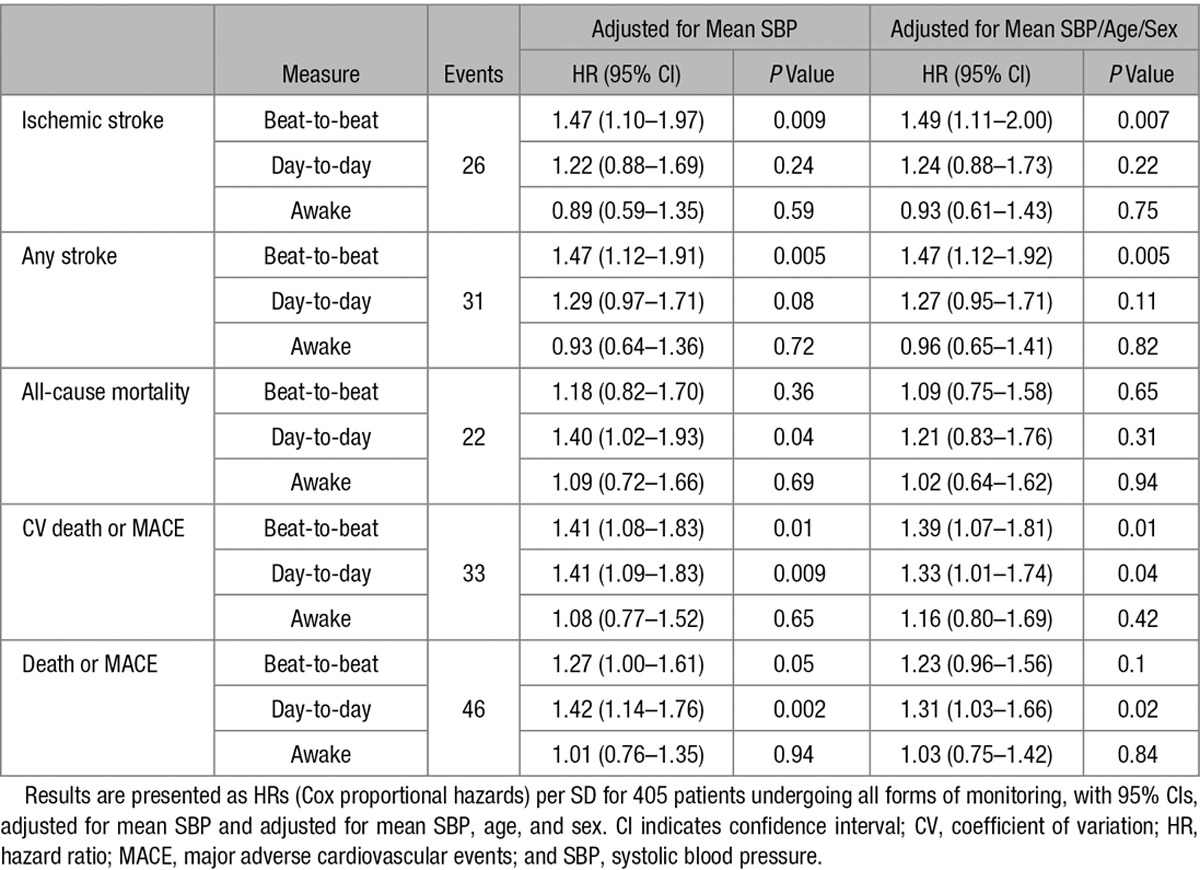
Risk of Cardiovascular Events During Follow-Up, According to Variability on Each Method of Blood Pressure Measurement

There was a significant increase in the absolute risk of recurrent stroke or all cardiovascular events across quartiles of BPV (Figure). Furthermore, beat-to-beat and day-to-day BPV were both moderately reproducible in 50 and 100 patients, respectively (intraclass correlation coefficient HBPM, 0.614; *P*<0.001; beat-to-beat, 0.503; *P*<0.001; Figure I in the online-only Data Supplement), resulting in a similar increase in the association between usual BPV on beat-to-beat and home monitoring after correction for regression dilution bias (Figure II in the online-only Data Supplement).

**Figure.**
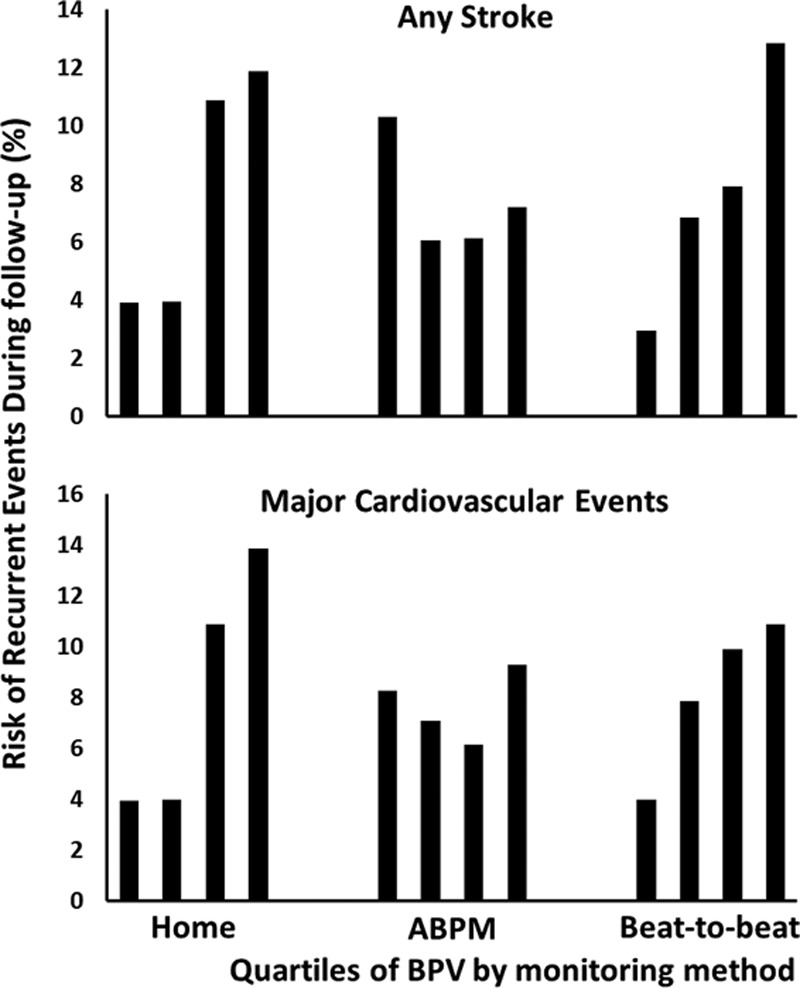
Absolute risks of recurrent stroke or major cardiovascular events by quartile of blood pressure variability (BPV) on each form of monitoring. The percentage risk of a recurrent stroke or major cardiovascular event (cardiovascular death, stroke, myocardial infarction, or acute peripheral vascular disease) during follow-up for 405 patients undergoing all forms of blood pressure monitoring is shown, subdivided by quartile of each method of monitoring. ABPM indicates ambulatory blood pressure monitoring.

In models including both beat-to-beat and HBPM BPV, beat-to-beat BPV was more predictive of the risk of recurrent stroke, whereas BPV on HBPM was more predictive of the risk of all cardiovascular events (Table V in the online-only Data Supplement). Similarly, mean BPV on beat-to-beat monitoring was significantly lower in patients unaffected by stroke than affected patients, whereas BPV on home monitoring was significantly lower compared with patients dying or experiencing outcome event (Table VI in the online-only Data Supplement).

Two hundred nineteen abstracts of 960 search responses were potentially relevant, with 34 articles reviewed in full. No study reported the risk of recurrent cardiovascular events per change in beat-to-beat BPV. As in our previous meta-analysis,^14^ the risk of a poor outcome after acute stroke was associated with both SBP variability (hazard ratio, 1.07 [0.9–1.2]) and DBP variability (hazard ratio, 1.33 [1.1–1.7]),^8,15^ whereas a reduced baroreceptor sensitivity was associated with poor outcome after stroke^9^ or myocardial infarction.^16,17^

## Discussion

BPV predicted the risk of recurrent stroke and all cardiovascular events on 5 minutes of beat-to-beat BP monitoring, with a ≈4-fold increase in risk between the lowest and highest quartile of the population, with broadly similar predictive power to BPV on day-to-day monitoring.

Residual visit-to-visit variability in BP on antihypertensive treatment has a poor prognosis, despite good control of mean BP, with an increased risk of stroke and all cardiovascular events,^1,2^ and benefits of some antihypertensive drugs in the prevention of stroke seem to be due partly to reduced variability in SBP.^3,4^ However, more rapid assessment and control of BPV would be clinically useful, especially in the acute phase after transient ischemic attack or stroke. BPV on home BP monitoring is also predictive of recurrent strokes and all cardiovascular events^5,6^ and can be assessed for days but still poses practical challenges in retrieving and analyzing equipment and readings. Our study shows that a rapid, 5-minute assessment of beat-to-beat BPV may have similar prognostic significance compared with HBPM. If affected by antihypertensive medication in the same way as visit-to-visit and HBPM BPV, beat-to-beat BPV could be a useful index to guide antihypertensive treatment decisions. However, we found only a weak correlation between BPV on different methods of measurement, yet they were independently related to outcomes. This is consistent with the weak relationship between within-visit and between-visit BPV in previous analyses of the ASCOT trial (Anglo-Scandinavian Cardiac Outcomes Trial).^2^ Therefore, BPV on beat-to-beat and home monitoring may well be a complementary measure, potentially reflecting different pathophysiological mechanisms leading to stroke.

We have demonstrated previously that home and beat-to-beat BPV are associated with a similar underlying physiological phenotype,^7^ including increased arterial stiffness, aortic pulsatility, reduced baroreceptor gain, and increased cardiovascular reactivity to stress. Furthermore, patients with an acute stroke have increased beat-to-beat BPV and reduced baroreceptor gain,^18^ which is associated with increased mortality^9^ and is partly dependent on stroke location.^19^ However, the precise mechanism by which BPV is associated with an increased risk of recurrent stroke is unclear. This may reflect either the effects of associated physiological indices (arterial stiffness, pulsatility, and cerebrovascular reactivity) or direct effects of beat-to-beat BPV itself. However, beat-to-beat BPV is a composite measure of multiple physiological processes, including irregular episodic components and rhythmic components related to breathing and to underlying autonomic rhythms (ie, low frequency oscillations at 0.04–0.15 Hz),^20^ and its prognostic significance may also reflect multiple pathophysiological processes.

Our study has some limitations. First, some patients were excluded because of poor-quality recordings, either because of poor peripheral circulation, excess ectopy, or atrial fibrillation during the recording. However, this reflects the strength of study, which included a consecutively recruited, unselected elderly population with acute events. Second, although statistical power to compare different measures of BPV was limited by the relatively small number of recurrent vascular events, the study is nevertheless the largest study of the prognostic significance of beat-to-beat SBP variability in patients with stroke. Third, BPV was estimated after initiation of antihypertensive treatment, which may affect BPV and its association with recurrent events. However, this also largely removes the confounding effects of inadequate mean BP control. Finally, repeated assessments for calculation of reproducibility of measures were performed after initiation of treatment. However, this would be expected to cause an underestimate of reproducibility.

Beat-to-beat BPV is, therefore, an appealing measure to increase our understanding of both physiology and cerebrovascular risk prediction, but its potential use in clinical practice is limited by the need for continuous BP monitoring, specialist analysis, a need for validation in other cohorts, and a lack of normative values and thresholds for pathologically relevant BPV. These questions will require further research before the application of beat-to-beat BPV in practice. Furthermore, its use will ultimately depend on its capacity to alter management through improved risk prediction or the identification and monitoring of a novel treatment target.

## Conclusions

Beat-to-beat BPV was a novel predictor of the risk of recurrent stroke and may be complementary to BPV on day-to-day home BP monitoring, may aid in risk stratification, and may help identify independently treatable mechanisms to reduce the risk of stroke.

## Acknowledgments

We acknowledge the use of facilities of the Acute Vascular Imaging Centre and the Cardiovascular Clinical Research Facility, University of Oxford.

## Sources of Funding

The Oxford Vascular Study is funded by the National Institute for Health Research (NIHR) Oxford Biomedical Research Centre, Wellcome Trust, Wolfson Foundation, British Heart Foundation, and the European Unions Horizon 2020 Programme (grant 666881, SVDs@target). P.M. Rothwell is in receipt of an NIHR Senior Investigator award. A.J.S. Webb is funded by a Wellcome Trust Clinical Research Development Fellowship and British Heart Foundation Project grant.

## Disclosures

None.

## Supplementary Material
