# Supplementary material for: Prognostic Significance of Blood Pressure Variability on Beat-to-Beat Monitoring After Transient Ischemic Attack and Stroke
Source: Stroke. 2017 Dec 11;49(1):62–7. doi: 10.1161/STROKEAHA.117.019107 (PMC5742536; doi:10.1161/STROKEAHA.117.019107)
Supplement: Supplementary file 1 [file str-49-062-s001.pdf]

ONLINE SUPPLEMENT

Prognostic significance of blood pressure variability on beat-to-beat monitoring after TIA and stroke

Alastair JS Webb[†] BMBCh DPhil, Sara Mazzucco[† χ] MD PhD, Linxin Li Dphil, *Peter M Rothwell[†] FMedSci

Supplemental Table I. Risk of cardiovascular events during follow up, according to variability on each method of BP measurement. Results are presented as hazard ratios (Cox Proportional Hazards) per standard deviation for all patients undergoing each form of monitoring, with 95% confidence intervals, unadjusted and adjusted for age and gender. Bt-to-bt = beat to beat BP variability; p-val=p value;

			Unadjusted		Adjusted for Age/Gender	
	**Measure**	**Ev**	**HR (95%CI)**	**p-val**	**HR (95%CI)**	**p-val**
**Ischaemic Stroke**	Bt-to-bt	23	1.51 (1.14 - 1.99)	0.004	1.51 (1.14 - 2.01)	0.005
	Day-to-day	22	1.21 (0.88 - 1.66)	0.24	1.25 (0.91 - 1.73)	0.17
	Awake	23	0.90 (0.61 - 1.32)	0.58	0.96 (0.65 - 1.44)	0.86
**Any Stroke**	Bt-to-bt	34	1.50 (1.16 - 1.94)	0.002	1.48 (1.14 - 1.93)	0.004
	Day-to-day	33	1.28 (0.97 - 1.68)	0.08	1.27 (0.96 - 1.69)	0.09
	Awake	33	0.95 (0.67 - 1.33)	0.75	1.00 (0.70 - 1.43)	0.99
**All cause mortality**	Bt-to-bt	24	1.26 (0.90 - 1.76)	0.18	1.12 (0.79 - 1.58)	0.53
	Day-to-day	28	1.33 (0.99 - 1.78)	0.06	1.09 (0.76 - 1.56)	0.65
	Awake	26	0.96 (0.66 - 1.40)	0.85	0.92 (0.61 - 1.38)	0.69
**CV Death or MACE**	Bt-to-bt	37	1.41 (1.09 - 1.82)	0.009	1.35 (1.04 - 1.75)	0.02
	Day-to-day	38	1.33 (1.04 - 1.70)	0.02	1.26 (0.98 - 1.63)	0.08
	Awake	38	0.95 (0.70 - 1.30)	0.76	1.04 (0.74 - 1.46)	0.83
**Death or MACE**	Bt-to-bt	53	1.32 (1.05 - 1.65)	0.02	1.23 (0.97 - 1.54)	0.08
	Day-to-day	55	1.34 (1.10 - 1.65)	0.005	1.22 (0.98 - 1.53)	0.08
	Awake	54	0.93 (0.71 - 1.21)	0.59	0.95 (0.72 - 1.27)	0.75

Supplemental Table II. Risk of cardiovascular events during follow up, according to variability on each method of BP measurement. Results are presented as hazard ratios (Cox Proportional Hazards) per 1% increase in CV for all patients undergoing each form of monitoring, with 95% confidence intervals, unadjusted and adjusted for age and gender. Bt-to-bt = beat to beat BP variability; p-val=p value;

			Unadjusted		Adjusted for Age/Gender	
	**Measure**	**Ev**	**HR (95%CI)**	**p-val**	**HR (95%CI)**	**p-val**
**Ischaemic Stroke**	Bt-to-bt	23	1.24 (1.07 - 1.43)	0.004	1.24 (1.07 - 1.43)	0.005
	Day-to-day	22	1.11 (0.93 - 1.33)	0.24	1.14 (0.95 - 1.36)	0.17
	Awake	23	0.97 (0.87 - 1.08)	0.58	0.99 (0.88 - 1.11)	0.86
**Any Stroke**	Bt-to-bt	34	1.23 (1.08 - 1.41)	0.002	1.23 (1.07 - 1.41)	0.004
	Day-to-day	33	1.15 (0.98 - 1.34)	0.08	1.15 (0.98 - 1.35)	0.09
	Awake	33	0.98 (0.89 - 1.08)	0.75	1.00 (0.90 - 1.11)	0.99
**All cause mortality**	Bt-to-bt	24	1.13 (0.95 - 1.34)	0.18	1.06 (0.89 - 1.27)	0.53
	Day-to-day	28	1.17 (0.99 - 1.39)	0.06	1.05 (0.85 - 1.29)	0.65
	Awake	26	0.99 (0.89 - 1.10)	0.85	0.98 (0.87 - 1.09)	0.69
**CV Death or MACE**	Bt-to-bt	37	1.19 (1.05 - 1.36)	0.009	1.17 (1.02 - 1.34)	0.02
	Day-to-day	38	1.18 (1.02 - 1.35)	0.02	1.14 (0.99 - 1.32)	0.08
	Awake	38	0.99 (0.9 - 1.08)	0.76	1.01 (0.92 - 1.11)	0.83
**Death or MACE**	Bt-to-bt	53	1.15 (1.03 - 1.29)	0.02	1.11 (0.99 - 1.25)	0.08
	Day-to-day	55	1.18 (1.05 - 1.33)	0.005	1.12 (0.99 - 1.27)	0.08
	Awake	54	0.98 (0.91 - 1.06)	0.59	0.99 (0.91 - 1.07)	0.75

Supplemental Table III. Risk of cardiovascular events during follow up, according to variability on each method of DBP measurement. Results are presented as hazard ratios (Cox Proportional Hazards) per SD increase in CV of DBP for all patients undergoing each form of monitoring, with 95% confidence intervals, unadjusted and adjusted for age and gender. Bt-to-bt = beat to beat BP variability; p-val=p value;

			Unadjusted		Adjusted for Age/Gender	
	**Measure**	**Ev**	**HR (95%CI)**	**p-val**	**HR (95%CI)**	**p-val**
**Ischaemic Stroke**	Bt-to-bt	31	1.27 (0.98 - 1.66)	0.07	1.24 (0.95 - 1.63)	0.11
	Day-to-day	31	0.97 (0.64 - 1.48)	0.90	1.00 (0.64 - 1.54)	0.98
	Awake	31	1.39 (0.99 - 1.95)	0.06	1.49 (1.03 - 2.16)	0.03
**Any Stroke**	Bt-to-bt	34	1.22 (0.96 - 1.55)	0.10	1.20 (0.95 - 1.52)	0.13
	Day-to-day	33	1.06 (0.77 - 1.46)	0.74	1.06 (0.76 - 1.48)	0.74
	Awake	33	1.14 (0.83 - 1.57)	0.42	1.16 (0.83 - 1.63)	0.39
**All cause mortality**	Bt-to-bt	24	1.20 (0.90 - 1.60)	0.21	1.09 (0.81 - 1.46)	0.58
	Day-to-day	28	1.42 (1.07 - 1.88)	0.02	1.28 (0.91 - 1.81)	0.16
	Awake	26	1.32 (0.94 - 1.85)	0.11	1.24 (0.88 - 1.76)	0.22
**CV Death or MACE**	Bt-to-bt	37	1.21 (0.96 - 1.52)	0.10	1.16 (0.92 - 1.46)	0.22
	Day-to-day	38	1.21 (0.92 - 1.58)	0.17	1.18 (0.89 - 1.57)	0.25
	Awake	38	1.27 (0.96 - 1.68)	0.10	1.36 (1.01 - 1.84)	0.04
**Death or MACE**	Bt-to-bt	53	1.16 (0.94 - 1.43)	0.17	1.09 (0.88 - 1.34)	0.44
	Day-to-day	55	1.23 (0.99 - 1.54)	0.07	1.16 (0.91 - 1.48)	0.22
	Awake	54	1.26 (0.99 - 1.59)	0.06	1.31 (1.02 - 1.67)	0.04

Supplemental Table IV. Risk of cardiovascular events during follow up, according to mean SBP on each method of BP measurement. Results are presented as hazard ratios (Cox Proportional Hazards) per SD increase in mean SBP for all patients undergoing each form of monitoring, with 95% confidence intervals, unadjusted and adjusted for age and gender. Bt-to-bt beat to beat BP variability; p-val=p value;

			Unadjusted		Adjusted for Age/Gender	
	**Measure**	**Ev**	**HR (95%CI)**	**p-val**	**HR (95%CI)**	**p-val**
**Ischaemic Stroke**	Bt-to-bt	23	1.55 (1.15 - 2.09)	0.004	1.56 (1.15 - 2.12)	0.005
	Day-to-day	22	1.61 (1.20 - 2.16)	0.002	1.58 (1.17 - 2.13)	0.003
	Awake	23	1.65 (1.19 - 2.30)	0.003	1.68 (1.21 - 2.35)	0.002
**Any Stroke**	Bt-to-bt	34	1.44 (1.08 - 1.92)	0.01	1.44 (1.07 - 1.93)	0.02
	Day-to-day	33	1.56 (1.19 - 2.06)	0.002	1.56 (1.17 - 2.07)	0.002
	Awake	33	1.60 (1.17 - 2.17)	0.003	1.59 (1.16 - 2.19)	0.004
**All cause mortality**	Bt-to-bt	24	1.47 (1.10 - 1.98)	0.01	1.43 (1.04 - 1.96)	0.03
	Day-to-day	28	1.40 (1.03 - 1.92)	0.03	1.64 (1.16 - 2.32)	0.005
	Awake	26	1.52 (1.08 - 2.15)	0.02	1.42 (0.95 - 2.12)	0.09
**CV Death or MACE**	Bt-to-bt	37	1.61 (1.24 - 2.08)	<0.001	1.59 (1.21 - 2.08)	<0.001
	Day-to-day	38	1.71 (1.33 - 2.19)	<0.001	1.73 (1.33 - 2.25)	<0.001
	Awake	38	1.67 (1.25 - 2.22)	<0.001	1.60 (1.19 - 2.16)	0.002
**Death or MACE**	Bt-to-bt	53	1.62 (1.31 – 2.00)	<0.001	1.56 (1.24 - 1.95)	<0.001
	Day-to-day	55	1.59 (1.28 - 1.97)	<0.001	1.66 (1.32 - 2.08)	<0.001
	Awake	54	1.69 (1.33 - 2.15)	<0.001	1.58 (1.23 - 2.04)	<0.001

Supplemental Table V. Relationship between BP variability on home or beat-to-beat monitoring and the risk of recurrent cardiovascular events, adjusted for both forms of monitoring. Results are presented as hazard ratios per standard deviation increase in BPV. Bt-to-bt= beat to beat BP variability; p-val=p value;

		Unadjusted		Adjusted for Age/Gender	
	**Measure**	**HR (95%CI)**	**p-val**	**HR (95%CI)**	**p-val**
**Ischaemic Stroke**	Bt-to-bt	1.41 (1.05 - 1.90)	0.02	1.43 (1.06 - 1.93)	0.02
	Day-to-day	1.18 (0.83 - 1.68)	0.37	1.19 (0.83 - 1.71)	0.33
**Any Stroke**	Bt-to-bt	1.38 (1.06 - 1.81)	0.02	1.39 (1.06 - 1.83)	0.02
	Day-to-day	1.25 (0.92 - 1.71)	0.16	1.24 (0.90 - 1.70)	0.19
**Death**	Bt-to-bt	1.22 (0.86 - 1.73)	0.27	1.13 (0.78 - 1.64)	0.53
	Day-to-day	1.40 (0.96 - 2.03)	0.08	1.21 (0.79 - 1.87)	0.38
**CV death or MACE**	Bt-to-bt	1.30 (1.01 - 1.67)	0.05	1.30 (1.01 - 1.68)	0.04
	Day-to-day	1.39 (1.06 - 1.82)	0.02	1.32 (1.00 - 1.74)	0.05
**Death or MACE**	Bt-to-bt	1.19 (0.96 - 1.49)	0.12	1.17 (0.93 - 1.47)	0.18
	Day-to-day	1.43 (1.15 - 1.78)	0.002	1.32 (1.05 - 1.67)	0.02

Supplemental Figure I. Agreement between BPV recorded on two separate occasions with either beat-to-beat or home monitoring. Panels A + B show scatter plots comparing the first and second recording of BPV over 5 minutes of beat-to-beat recording (A) or 1 week of day-to-day home recording (B). Panels C+D show the equivalent Bland-Altman plots for agreement between the two measurements.

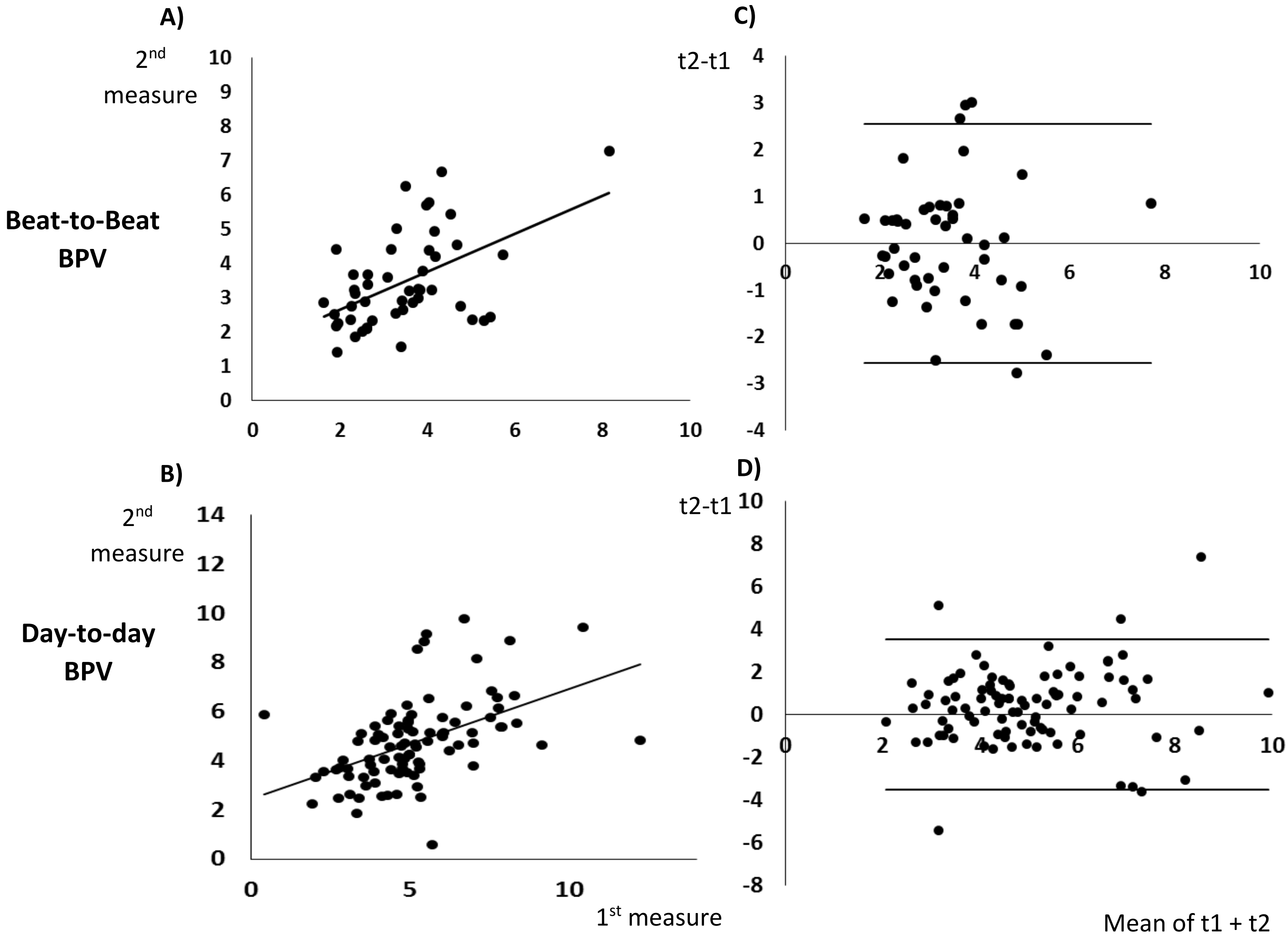

Supplemental Figure II. Effect of adjustment for regression to the mean on the relationship between BPV on beat-to-beat or home BP monitoring and the risk of recurrent cardiovascular events or death. Hazard ratios for the risk of recurrent events for each quartile of BPV on beat-to-beat or home monitoring relative to the lowest quartile are shown, before (A) and after (B) adjustment for regression to the mean. Difference between the mean BPV for each quartile and the population mean was adjusted by the intraclass correlation coefficient from repeatability studies.

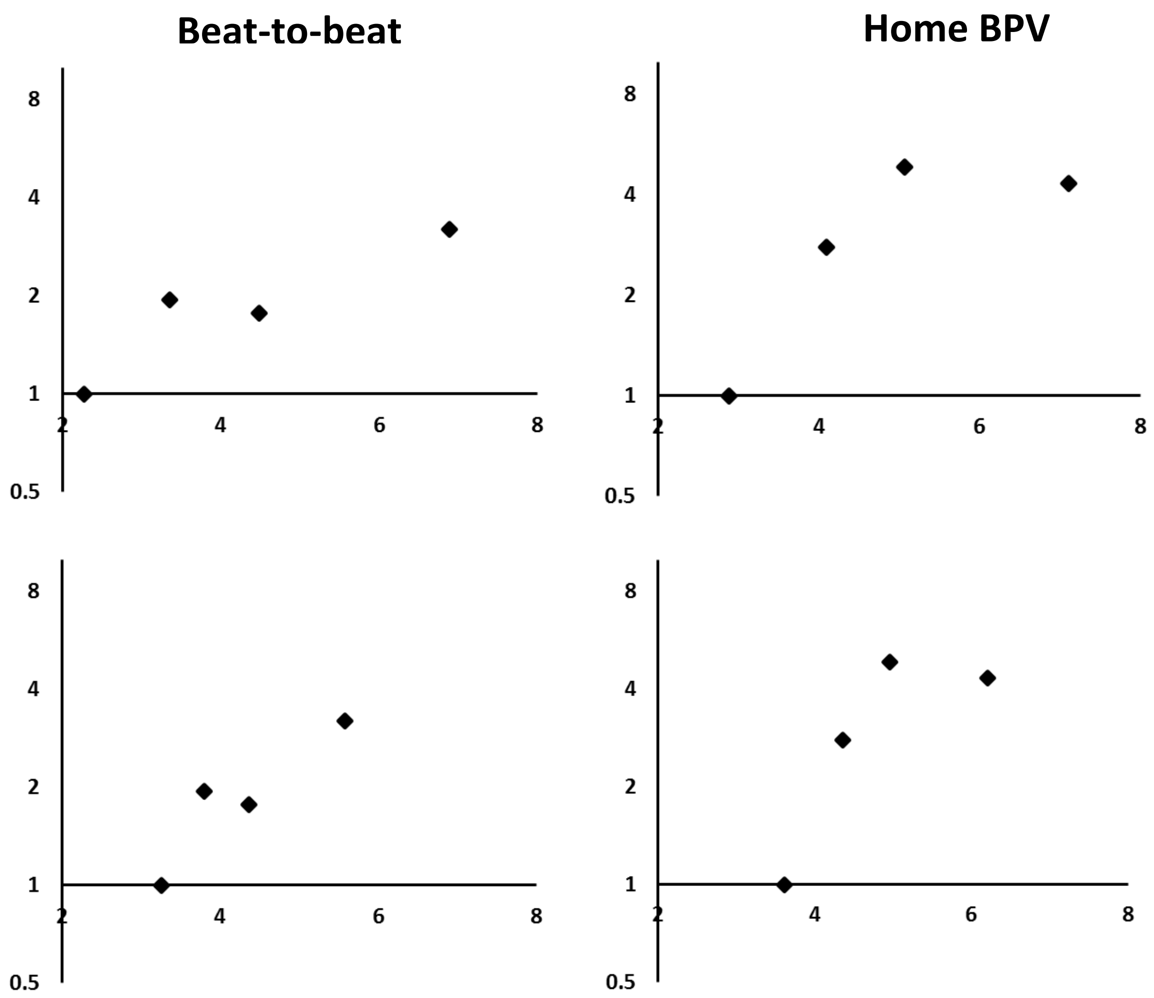

Supplemental Table VI. Differences in mean SBPV on beat-to-beat and home day-to-day monitoring for patients experiencing recurrent events. Results are presented as mean (standard deviation) for patients affected or unaffected by each outcome event. P-values (p-val) are presented for t-tests. MACE = Major adverse cardiovascular events, CVS = cardiovascular, CV = coefficient of variation.

Event	Beat-to-beat BPV			Home BPV		
	Unaffected	Affected	p-val	Unaffected	Affected	p-val
Ischaemic Stroke	4.68 (2.4)	6.24 (3.6)	0.001	4.75 (1.8)	5.19 (1.7)	0.21
Any Stroke	4.66 (2.4)	6.16 (3.6)	0.001	4.74 (1.8)	5.34 (1.6)	0.06
All cause mortality	4.70 (2.5)	6.14 (3.3)	0.006	4.73 (1.8)	5.45 (1.5)	0.03
CV Death or MACE	4.66 (2.4)	5.97 (3.5)	0.002	4.71 (1.7)	5.46 (1.9)	0.01
Death or MACE	4.60 (2.4)	5.98 (3.5)	0.03	4.68 (1.8)	5.44 (1.7)	0.02
